# Impact of Circulating Cholesterol Levels on Growth and Intratumoral Androgen Concentration of Prostate Tumors

**DOI:** 10.1371/journal.pone.0030062

**Published:** 2012-01-18

**Authors:** Elahe A. Mostaghel, Keith R. Solomon, Kristine Pelton, Michael R. Freeman, R. Bruce Montgomery

**Affiliations:** 1 Division of Clinical Research, Fred Hutchinson Cancer Research Center, Seattle, Washington, United States of America; 2 Department of Medicine, University of Washington, Seattle, Washington, United States of America; 3 Department of Orthopaedic Surgery, Children's Hospital Boston, Boston, Massachusetts, United States of America; 4 Department of Urology, Children's Hospital Boston, Boston, Massachusetts, United States of America; 5 Department of Orthopaedic Surgery, Harvard Medical School, Boston, Massachusetts, United States of America; 6 Department of Surgery, Harvard Medical School, Boston, Massachusetts, United States of America; 7 Department of Biological Chemistry and Molecular Pharmacology, Harvard Medical School, Boston, Massachusetts, United States of America; Thomas Jefferson University, United States of America

## Abstract

Prostate cancer (PCa) is the second most common cancer in men. Androgen deprivation therapy (ADT) leads to tumor involution and reduction of tumor burden. However, tumors eventually reemerge that have overcome the absence of gonadal androgens, termed castration resistant PCa (CRPC). Theories underlying the development of CRPC include androgen receptor (AR) mutation allowing for promiscuous activation by non-androgens, AR amplification and overexpression leading to hypersensitivity to low androgen levels, and/or tumoral uptake and conversion of adrenally derived androgens. More recently it has been proposed that prostate tumor cells synthesize their own androgens through *de novo* steroidogenesis, which involves the step-wise synthesis of androgens from cholesterol. Using the *in vivo* LNCaP PCa xenograft model, previous data from our group demonstrated that a hypercholesterolemia diet potentiates prostatic tumor growth via induction of angiogenesis. Using this same model we now demonstrate that circulating cholesterol levels are significantly associated with tumor size (R = 0.3957, p = 0.0049) and intratumoral levels of testosterone (R = 0.41, p = 0.0023) in LNCaP tumors grown in hormonally intact mice. We demonstrate tumoral expression of cholesterol uptake genes as well as the spectrum of steroidogenic enzymes necessary for androgen biosynthesis from cholesterol. Moreover, we show that circulating cholesterol levels are directly correlated with tumoral expression of CYP17A, the critical enzyme required for *de novo* synthesis of androgens from cholesterol (R = 0.4073, p = 0.025) Since hypercholesterolemia does not raise circulating androgen levels and the adrenal gland of the mouse synthesizes minimal androgens, this study provides evidence that hypercholesterolemia increases intratumoral *de novo* steroidogenesis. Our results are consistent with the hypothesis that cholesterol-fueled intratumoral androgen synthesis may accelerate the growth of prostate tumors, and suggest that treatment of CRPC may be optimized by inclusion of cholesterol reduction therapies in conjunction with therapies targeting androgen synthesis and the AR.

## Introduction

Prostatic malignancies, benign prostatic hyperplasia, and normal prostate tissues lose homeostatic control over cholesterol level with age, synthesize cholesterol at a high rate, and thereby accumulate excess levels of cholesterol [1], [2], [3]. The overall consequence of this cholesterol accumulation on prostate physiology is unknown, but a role for high levels of serum cholesterol in PCa incidence and progression has been suggested by a number of epidemiological and pre-clinical studies [4], [5], [6], [7], [8], [9]. While high fat/high cholesterol ‘Western’ diets have been linked to PCa incidence and progression in some reports, a role for specific dietary components in disease progression has not been clearly established [10], [11]. Studies examining groups of nutritional components eaten together suggest that diets with a high content of cholesterol-rich, processed and/or red meat may be associated with higher PCa incidence [12], [13]. In addition, observational studies of cholesterol-lowering drug use (i.e. HMG-CoA reductase inhibitors, aka statins) and cancer incidence, which include large numbers of PCa patients and a substantial number with advanced disease, show an inverse association between statin use and PCa incidence and/or progression, including a significant reduction in risk of advanced disease with long term statin use [14], [15], [16], [17], [18], [19], [20], [21], [22], [23], [24]. Although not all studies support this association [25], [26], [27], the preponderance of evidence suggests that cholesterol plays a role in PCa progression, with its most likely role being a factor in the progression to advanced disease.

We have demonstrated that hypercholesterolemic diets stimulate growth of LNCaP human PCa xenografts [9], [28]. Tumors in the hypercholesterolemic environment accumulated more cholesterol in their membranes, exhibited lower levels of apoptosis, had enhanced activation of Akt (a kinase linked to aggressive PCa) [29], [30], [31], [32], and were more angiogenic [9], [28]. We also demonstrated that a hypocholesterolemic diet has the opposite effect, inhibiting the growth of prostatic tumors. In explaining these results, we hypothesized that cholesterol might directly contribute to tumor growth by altering signal transduction pathways [1], [28], [33], consistent with the role of cholesterol in organizing liquid ordered membrane domains [34]. But other explanations for the effect of hypercholesterolemia on PCa risk warrant careful consideration. In particular, one important new hypothesis is that cholesterol affects PCa growth by serving as a precursor for intratumoral androgen synthesis.

Androgen Deprivation Therapy (ADT) is the primary treatment strategy for advanced metastatic PCa [35], [36], [37]. However, despite initial efficacy as well as maintenance of castrate levels of circulating androgens, progression to castration resistant prostate cancer (CRPC) invariably occurs [35], [36]. Theories regarding development of the castration-resistant phenotype include: 1) gene amplification and/or mutation of the AR, allowing the receptor to be sensitive to low levels of androgen [38], [39], [40], [41], [42], [43], [44]; 2) residual androgen production from the adrenals [45]; and 3) promiscuous receptor-ligand interactions [40], [46].

More recently, several lines of evidence are converging on the hypothesis that PCa cells synthesize their own androgens, including in the castrate environment [47], [48], [49], in sufficient quantities to activate the AR. Prior studies [47] have shown that all of the enzymes necessary for *de novo* steroidogenesis are expressed in LNCaP tumor xenografts, and that androgen-starved PCa cells are capable of synthesizing DHT from acetic acid, suggesting that the entire mevalonate-steroidogenic pathway is intact in this model system [47]. In a prior report, we demonstrated that the full complement of enzymes comprising the steroidogenic pathways are present in a significant subset of human primary and metastatic PCas examined [49], implying that *de novo* androgen synthesis is not merely an experimental phenomenon, but rather a potential underlying cause of disease progression in the hormone-repressed state. In addition, this and other studies established that serum and tissue steroid hormone concentrations are not correlated, indicating that detailed analysis of tissue hormones is necessary to evaluate the effects of cholesterol on tissue steroidogenesis.

In the current study using tumors partially described previously [9], we follow-up on our prior reports demonstrating that a hypercholesterolemic diet promotes, while hypocholesterolemic diet retards, the growth of prostate tumors *in vivo*, by demonstrating that the level of circulating cholesterol is associated with tumor growth and the intratumoral level of androgens (especially testosterone (T)). This is the first study of any kind demonstrating that circulating cholesterol levels influence tumor androgen levels and the first to demonstrate that pharmacological lowering of cholesterol reduces the level of tumor androgens. These new data suggest that cholesterol-based management of prostate cancer progression may be an effective strategy at reducing tumor growth in patients.

## Materials and Methods

### Mice and tumor xenografts

All procedures were done in compliance with Children's Hospital Boston's animal care and use policies; protocol 07-11-1416R covering these procedures was approved by the IACUC of Children's Hospital Boston. 5 week old SCID mice obtained from the Massachusetts General Hospital were fed a low fat/no cholesterol diet (LFNC) (Research Diets, New Brunswick, NJ diet # D12102). After two weeks, blood was drawn from the saphenous tail vein and the serum cholesterol concentration measured using the Infinity Cholesterol Liquid Stable Reagent (Thermo Electron Corp.). The mice were then divided into high fat/high cholesterol diet (HFHC) (Research Diets, diet # D12108) and LFNC diet groups ± ezetimibe (30 mg/kg(mouse weight)/day; Schering-Plough, New Brunswick, NJ, added to powdered food) and the mice continued on these diets for two weeks prior to tumor implantation. Mice were not fed a libitum, but instead diets were fed to mice isocalorically with each mouse receiving 17.32 kcal/day. These diets do not produce any difference in mouse body weight between the cohorts [9] (data not shown).

Xenografts were initiated by injecting LNCaP (2×10^6^ per site) with 1∶1 volume of Matrigel (BD BioSciences, San Jose, CA) into the 4 dorsal quadrants of each mouse. To eliminate any injection bias, mice were randomized prior to implantation and the implanter was blinded to the mouse cohort assignment. Tumors were measured daily from the appearance of the first palpable tumors and the mice were sacrificed prior to achieving the maximum tumor burden (≈13 days post implantation). Terminal bleeds were taken (cardiac puncture) for serology (triglyceride, bilirubin and other liver function tests were performed in the Dept. of Laboratory Medicine, Children's Hospital Boston, androgen levels were determined by a testosterone EIA, Diagnostic Systems Laboratories, Webster, TX). Tumors were removed, measured, weighed and either placed in OCT solution (Tissue-Tek, Torrance, CA) or snap frozen. For the purpose of analysis, each of the 4 tumors per mouse was regarded as a separate entity. There was no correlation of tumor size amongst the 4 tumors and there was no evidence of a dominant effect of one tumor on the others. A prior report by our group contains details on aspects of the growth and histology of these tumors [9].

### Cell culture

LNCaP human prostate cancer cells (American Type Culture Collection, Manassas, VA), which do not express either caveolin [33] or PTEN [50] were cultured in RPMI (Invitrogen, Carlsbad, CA) media supplemented with 10% FBS and 1% Penicillin/Streptomycin in 5% CO_2_ at 37°C.

### Tumor androgen analysis

30–50 mg pieces of snap frozen randomly chosen tumors, ≈13 from each diet cohort, were analyzed for tissue androgen levels by mass spectrometry (MS) using methods we have recently described [51]. This procedure resulted in a lower limit of quantitation of 1 pg per sample for T and DHT respectively. Intra-assay coefficients of variation generated using human serum for high, mid and low-range samples were 3.5, 3.1 and 3.8% for T and 6.3, 4.3 and 15.8% for DHT respectively.

### RNA isolation and quantitative RT-PCR

RNA was isolated and prepared for quantitative PCR (qRT-PCR) as previously described [49]. cDNA was generated in a random-primed reverse transcription reaction, and qRT-PCR reactions were performed in triplicate using an Applied Biosystems 7700 sequence detector with 5 ng of cDNA, 1 µM of each primer pair and SYBR Green PCR master mix (Applied Biosystems, Foster City, CA). Specificity of amplification was assessed based on melting point of the dissociation curve. Primer sequences were as previously published [49] as well as including the following: *SR-B1* Forward CCTCACAGGGTCCCTCAGATTAT, Reverse TTCCAGTAGAAAAGGGTCACAGG; *LDLR* Forward TTGCCTCTGAAATGCCTCTTCT, Reverse ATCATTCTCCCAAAAAGCGTTG; *STARD3* Forward GAAAGAGTCTGGGACCCTTGTTG, Reverse CCTGGGAGAGGCAGAGATTCAT; and *CYB5A* Forward AGATTCAGAAGCACAACCACAGC, Reverse TTAAAACTTCTTCCCCACCAGGA. Although no new sequence data was generated, all new data has been deposited in GenBank.

### Statistics

Unpaired two sample t-tests were used to compare differences in samples above or below the median. One way ANOVA was used to assess variance across quartiles, with a post test for linear trend between mean values in each quartile. Linear regression was used for assessment of correlations. In all cases, p values<0.05 were considered significant. For analysis of qRT-PCR data, the mean cycle threshold (Ct) for each gene was normalized to expression of the housekeeping gene RPL13A in the same sample (delta Ct).

## Results

**Figure 1:**
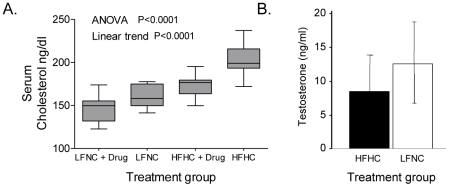
Serum cholesterol and testosterone levels in murine cohorts receiving cholesterol targeted treatment. (**A**) Mean cholesterol levels (with standard deviation and range) in mice randomized to 12 weeks of treatment with the indicated combinations of a low fat/no cholesterol diet (LFNC) or high fat/high cholesterol diet (HFHC) ± ezetimibe (drug). One way ANOVA with a post test for linear trend was used to compare values in the four treatment groups. P values<0.05 were considered significant. (**B**) Measurement of serum testosterone levels by ELISA in mice receiving the indicated HFHC or LFNC diet (n = 10/group). Data are presented as testosterone (T) levels (ng/ml) vs. diet group ± SE. Differences between mice fed the two diets were not statistically significant (unpaired two sample t-test).

We have created an innovative isocaloric diet approach allowing us to determine the specific effects of cholesterol in mice, by generating 4 murine cohorts, each with a different serum cholesterol level (≈140, 160, 180 & 200 mg/dL; **Fig. 1A**). As we have shown [9], this approach permits us to study hyper- and hypocholesterolemia simultaneously without affecting liver function, insulin levels, animal weight, or circulating steroid hormone levels. The basic design of our cholesterol-targeting approach combines a diet regimen with a pharmaceutical agent, ezetimibe (Zetia), an FDA approved drug that specifically blocks cholesterol uptake in the intestine, thereby lowering serum cholesterol levels. In this scheme we use a low fat/no cholesterol diet (LFNC) and a high fat/high cholesterol diet (HFHC) (w/o sodium cholate) ± ezetimibe (30 mg/kg/d added to powdered food). Ezetimibe is a specific antagonist of NPC1L1, the *bona fide* gut cholesterol transporter [52], [53], [54], [55]. Ezetimibe has no known target other than NPC1L1, and NPC1L1 is expressed only in the intestine and in hepatocytes (in humans), but not by tumor cells.

As we previously reported [9] mice fed the HFHC diet developed larger tumors (weight and volume), whereas mice fed the LFNC diet had smaller tumors. The addition of ezetimibe to either diet reduced tumor size. The combination of the LFNC diet+ezetimibe had the most significant effect on tumor growth (vs. the HFHC diet w/o ezetimibe). Statistical evaluation demonstrated that both the diet (p = 0.048) and ezetimibe (p = 0.035) produced significant independent, additive, but not synergistic, effects on tumor growth (both volume and weight). Serology showed no liver dysfunction, no statistical differences in triglyceride (TG) levels (trending higher in the HFHC cohort), & no statistical differences in insulin or IGF-1 levels (not shown). Importantly, the HFHC diet did not increase serum T levels over the LFNC diet (**Fig. 1B**), as expected, since circulating cholesterol does not influence serum androgen levels [56].

Our prior analysis only concerned whether HFHC and LFNC diets ± ezetimibe were associated with tumor growth, not whether tumor growth was a function of circulating cholesterol, per se. In our current analysis we unbound the groups and determined more precisely the relationship between circulating cholesterol level and tumor characteristics, as cholesterol level does not correspond directly to diet group, i.e. while HFHC raised cholesterol levels for the whole group, not every animal was in the highest cholesterol quartile, and similarly, while the LFNC diet+ezetimibe diet cohort had the lowest cholesterol level as a group not every animal in the cohort was in the lowest cholesterol quartile. Thus, while the HFHC cohort corresponds well to the highest quartile of cholesterol (9/10 were in Q4), the other quartiles contain a mixture of animals/tumors from the other 3 treatment groups (data not shown).

**Figure 2:**
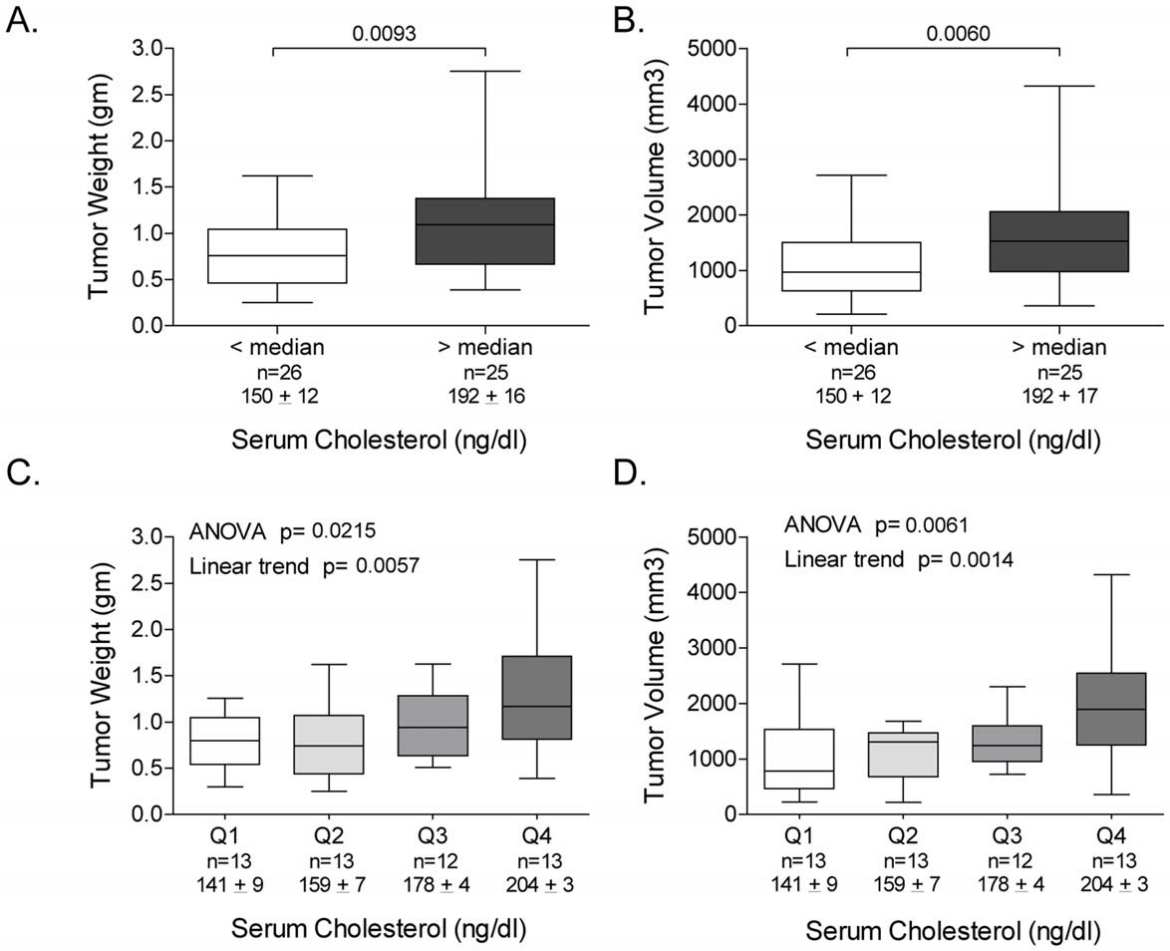
Growth of LNCaP xenograft tumors in relation to serum cholesterol levels. Mean tumor weight (**A**) and volume (**B**) (with standard deviation and range) in mice with cholesterol levels above or below the median serum cholesterol level. P values from unpaired two sample t-tests. Mean tumor weight (**C**) and volume (**D**) (with standard deviation and range) in mice grouped by quartile of serum cholesterol levels. P values from one way ANOVA of mean values in the four quartiles, with a post test for linear trend. The mean cholesterol levels in tumors above or below the median, or in each quartile of cholesterol, and the number of mice in each group, are indicated below the x-axis in each graph.

To determine the relationship between circulating cholesterol levels and tumor growth we analyzed tumor data from the 4 cohorts in aggregate, and plotted tumor size (weight and volume) vs. serum cholesterol level. Both tumor weight (**Fig. 2A**) and volume (**Fig. 2B**) were significantly larger in animals with levels of circulating cholesterol above vs. below the median, and demonstrated significant linear trends over the quartiles of cholesterol (**Fig. 2C,D**, respectively). For example, tumor weight ranged from a mean of 0.79±0.3 gm in tumors in the lowest quartile of serum cholesterol (Q1) to 1.32±0.6 gm in the highest quartile (Q4; p = 0.0057 for linear trend), consistent with the hypothesis that higher cholesterol levels promote tumor growth.

Although modulating serum cholesterol did not alter circulating T levels (**Fig. 1B**), LNCaP tumor cells express all the enzymes required to synthesize androgens from cholesterol (i.e. *de novo* steroidogenesis), giving us the opportunity to determine if circulating cholesterol levels influence intratumoral androgen levels. Snap frozen samples from 52 LNCaP tumors (∼n = 13 in each treatment group) were analyzed by MS for levels of T and DHT. Overall, mean tumor T levels were 2.71±1.66 pg/mg (range 0.44–7.39 pg/mg), and mean DHT levels were 0.61±0.16 pg/mg (range 0.25–0.96 pg/mg). This ratio of T to DHT is similar to that observed by Locke et al in a previous study of castration sensitive LNCaP tumors prior to castration [47].

**Figure 3:**
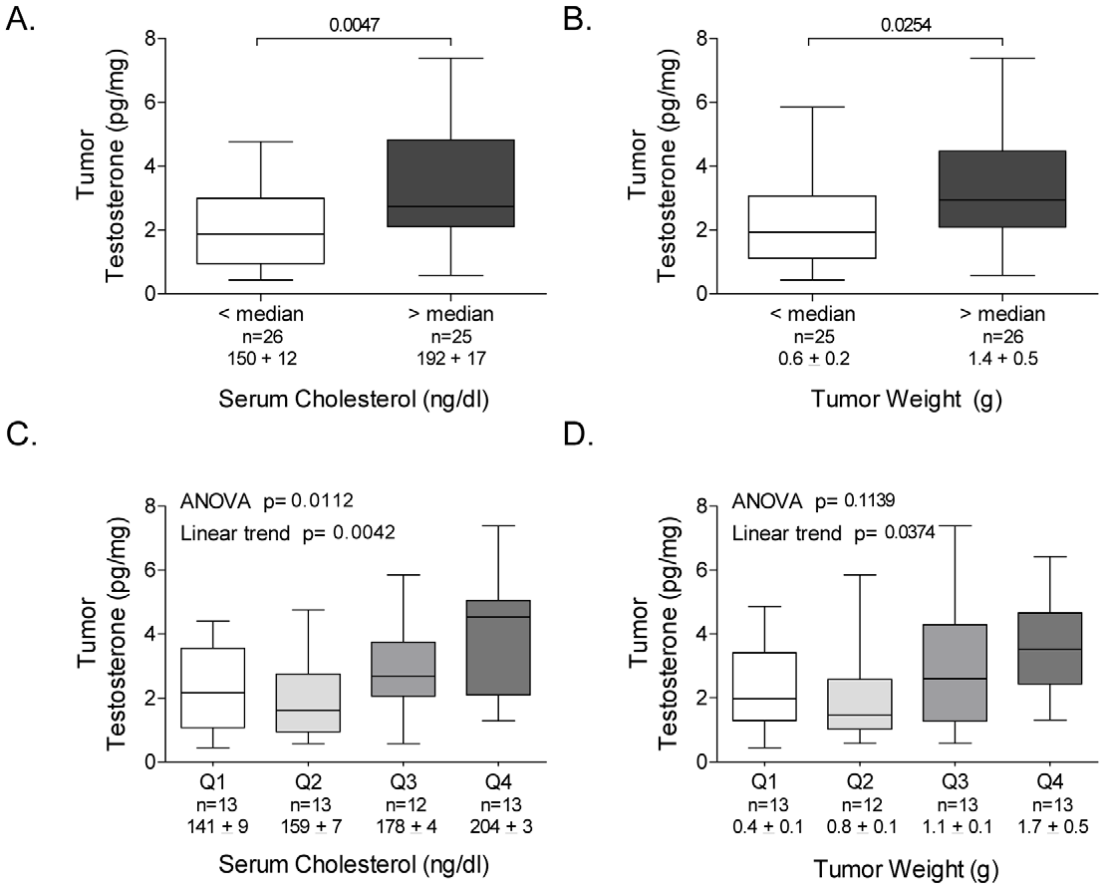
Tumor testosterone levels in LNCaP xenografts in relation to serum cholesterol levels and tumor weight. Mean testosterone levels (with standard deviation and range) measured by mass spectrometry in tumors from mice with cholesterol levels above or below the median cholesterol level (**A**), or in tumors above or below the median weight (**B**). The mean cholesterol levels and tumor weights in tumors above or below the median, and the number of mice in each group, are indicated below the x-axis in each graph. P values from unpaired two sample t-tests. Mean testosterone levels in tumors from mice grouped by quartile of serum cholesterol (**C**), or in tumors grouped by quartile of tumor weight (**D**). P values from one way ANOVA of mean values in the four quartiles, with a post test for linear trend. The mean cholesterol levels and tumor weights in tumors in each quartile of cholesterol or weight, and the number of mice in each group, are indicated below the x-axis in each graph.

**Figure 4:**
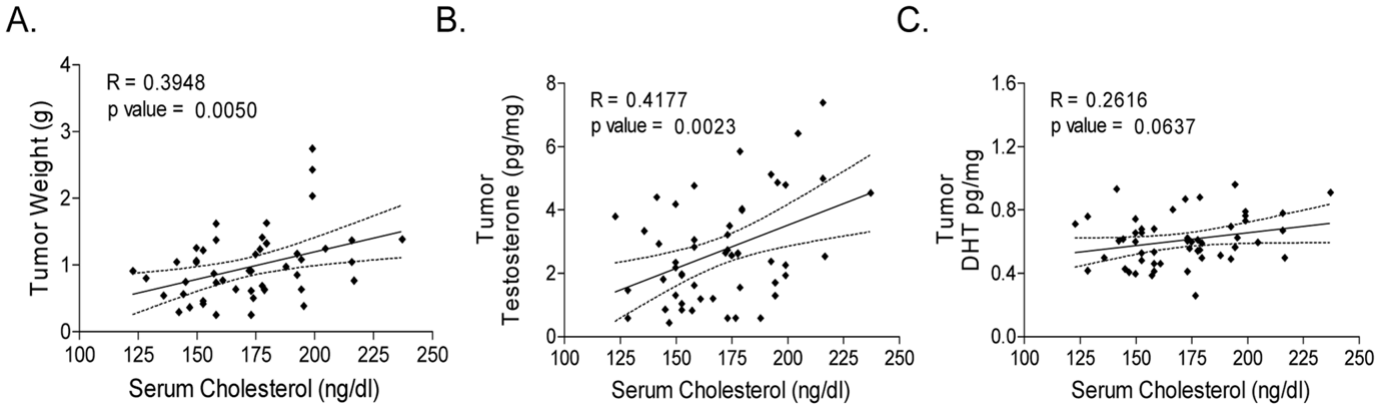
Correlation of serum cholesterol levels with tumor weight, tumors androgens and expression of CYP17A in LNCaP xenografts. The correlation of serum cholesterol levels with tumor weight (**A**), testosterone (**B**) and DHT (**C**) in aggregated tumors from all four treatment cohorts (n = 52). Tumor androgens were determined by mass spectrometry. Correlation coefficients and p values from linear regression analysis.

To explore the relationship between cholesterol, tumor androgens, and tumor growth we analyzed all the tumor data in aggregate, and plotted tumor T vs. tumor weight or tumor cholesterol. As demonstrated in **Fig. 3**, mean tumor T levels were higher in animals with circulating cholesterol levels above compared to below the median (**Fig. 3A**; 2.1±1.2 vs. 3.4±1.8, p = 0.0047), as well as higher in the larger tumors (**Fig. 3B**; 2.2±1.4 vs. 3.3±1.8, p = 0.025). Similarly, tumor T levels demonstrated significant linear trends over the quartiles of both serum cholesterol (**Fig. 3C**; p = 0.0042) and tumor weight (**Fig. 3D**; p = 0.037). Cholesterol quartile analysis indicated that DHT levels also trended higher as cholesterol level rose, but did not reach statistical significance (Figure S1). Notably, linear regression demonstrated significant correlations between serum cholesterol and tumor weight (**Fig. 4A**, R = 0.3957, p = 0.0049), serum cholesterol and tumor T (**Fig. 4B**, R = 0.4179, p = 0.0023), and near significance for serum cholesterol and tumor DHT (**Fig. 4C**, R = 0.2613, p = 0.06).

**Figure 5:**
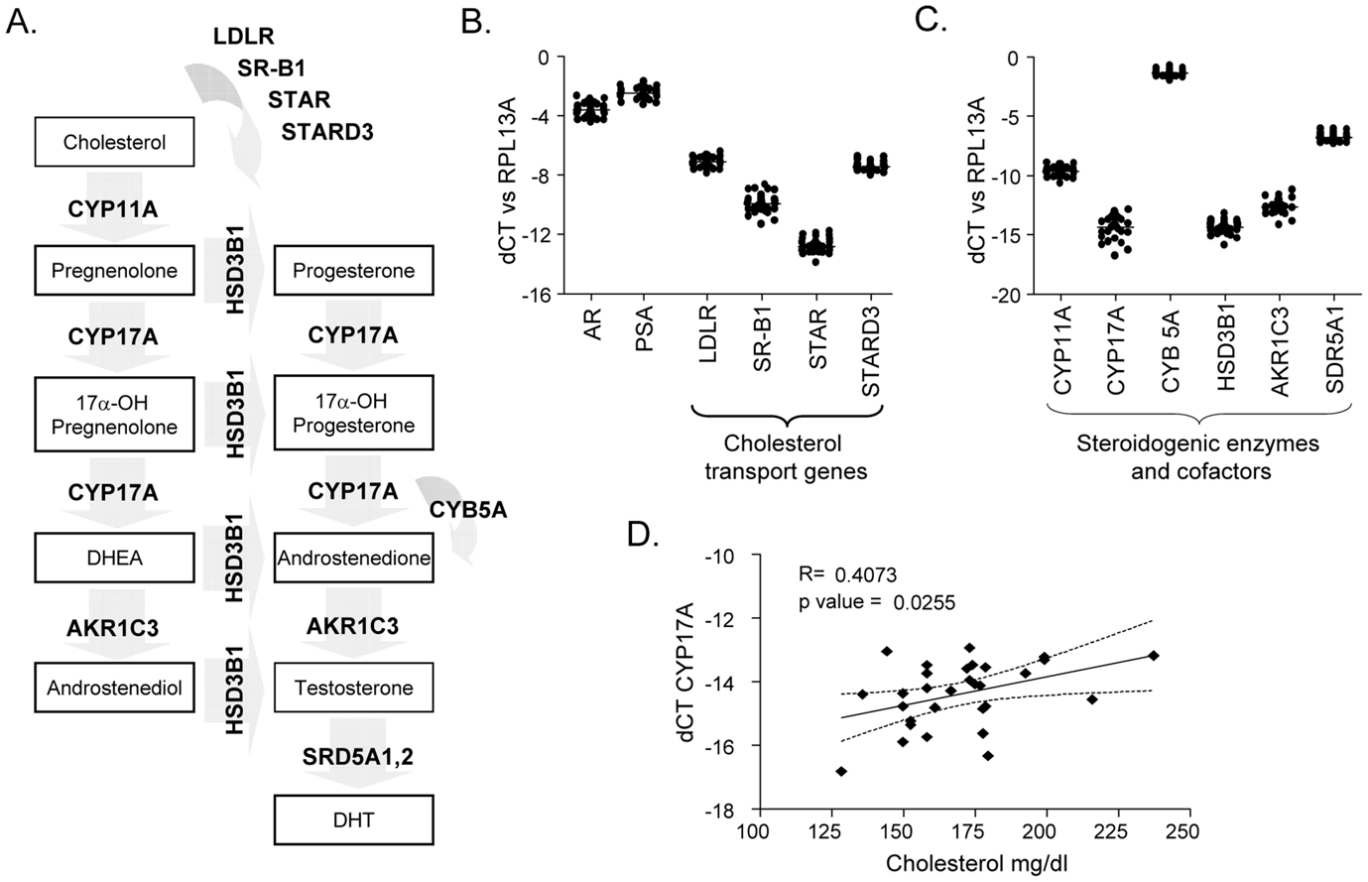
Expression of steroidogenic enzymes necessary for *de novo* synthesis of androgens from cholesterol in LNCaP xenografts. (**A**) Enzymes and intermediates in the steroid bio-synthetic pathway leading from cholesterol to the formation of testosterone and DHT. LDLR and SR-B1 mediate cholesterol uptake; STAR and STARD3 mediate transport of cholesterol across the mitochondrial membrane where steroidogenesis is initiated. CYB5A is an important cofactor for the lyase activity of CYP17A. (**B**) Transcript profiling of tumors from all 4 treatment groups by qRT-PCR for the androgen receptor (AR), the androgen regulate gene PSA, and genes involved in cholesterol transport. (**C**) Transcript profiling for the expression of steroidogenic genes and the CYB5 cofactor. The mean cycle threshold (CT) for detection of each transcript was normalized to expression of the housekeeping gene RPL13A in the same sample (delta or dCT). (**D**) The correlation of serum cholesterol levels with transcript expression of CYP17A as measured by qRT-PCR. Correlation coefficients and p values from linear regression analysis.

To determine whether the xenograft tumors expressed the steroidogenic genes necessary to generate T and DHT from circulating cholesterol, we profiled the tumors for expression of transcripts encoding each enzyme in the steroid bio-synthetic pathway, as well as several genes related to cholesterol uptake and mitochondrial transport (**Fig. 5A**). The majority of tumors had demonstrable expression of cholesterol transport genes (**Fig. 5B**), as well as the critical enzymes required for de novo steroidogenesis (**Fig. 5C**). While the majority of these genes did not demonstrate a significant correlation with cholesterol level, their expression levels are consistent with values previously reported in human PCa metastases with elevated tumor androgens [49], and in PCa xenograft models [57]. Of particular importance, serum cholesterol levels were significantly correlated with tumoral expression of 17 α-hydroxylase (CYP17A), the central enzyme required for *de novo* synthesis of androgens from cholesterol (**Fig. 5D**, R = 0.4073, p = 0.0255). Serum cholesterol levels also demonstrated a significant inverse correlation with the LDLR (low density lipoprotein receptor; R = −0.512, p = 0.0009), demonstrating that although these tumors accumulated cholesterol, certain normal feedback mechanisms regulating cholesterol uptake were functional (data not shown).

## Discussion

30 years ago Geller et al [58] first reported that sufficient androgen to drive the AR remained in the prostate after castration [58]. Indeed, the positive results of a phase III trial using the CYP17 inhibitor, abiraterone, in men with CRPC demonstrates that for many men, CRPC remains androgen-driven [59], [60]. Modern studies also suggest that PCa cells in men receiving ADT have androgen levels high enough to activate the AR despite castrate levels of circulating androgens [59], [61], [62], [63], [64], [65], [66]. The mechanisms responsible for maintenance of functional levels of T and DHT in CRPC tumors are unknown, but have been hypothesized to reflect the uptake and conversion of adrenal androgens or *de novo* synthesis of T from cholesterol or earlier precursors.

Here we show that elevations in serum cholesterol are directly correlated with tumor size, tumor T levels and expression of the key steroidogenic enzyme, CYP17A, in the castration sensitive LNCaP xenograft model, suggesting that one source of intratumoral androgen in prostate tumors is via *de novo* synthesis from circulating cholesterol. In mice the adrenal glands express little to no CYP17A, the essential enzyme required to convert pregnenolone to androstenedione, and consequently produce little to no androgen or androgen precursors (e.g. androstenediol) [47], [67]. The relative lack of adrenal contribution to androgen levels in this model, and the absence of diet-induced changes in circulating T (**Fig. 1**) suggest that the primary mechanism of the diet-induced increase in tumor androgen concentrations is due to *de novo* intratumoral synthesis from exogenous cholesterol.

In our prior report on the growth-promoting effects of cholesterol on prostate tumors [9] we determined that one effect of hypercholesterolemia was to increase tumor angiogenesis, which was associated with a substantial decrease in the expression of thrombonspondin-1 (TSP1) an angiogenesis inhibitor. Interestingly, TSP1 expression is suppressed by androgen [68]. Patients receiving androgen ablation therapy (ADT) were reported to exhibit a substantial increase in TSP1 expression in comparison to prostate cancers in patients not receiving ADT. The present data demonstrating that hypercholesterolemia increases androgen expression may explain why hypercholesterolemia also reduces TSP1 expression and increases angiogenesis, thereby facilitating tumor growth.

It is of particular interest that a targeted low fat, low cholesterol diet was able to modulate tumor androgen profiles in the hormonally intact environment in which tumor growth is not dependant on non-testicular sources of androgen. It appears that once a tumor has steroidogenic potential (as has been demonstrated for LNCaP tumors) [47], cholesterol acts as a critical, yet modifiable, factor for tumor growth through up-regulating androgen synthesis, even when intratumoral androgen synthesis is not absolutely required. A number of studies have shown that use of cholesterol-lowering statin drugs, which inhibit the rate limiting enzyme in cholesterol synthesis, HMG-CoA reductase, reduce the risk of advanced PCa, especially when used for more than 5 years, and usually many years prior to any evidence of cancer [15], [17], [18], [19], [20], [22], [23], [24]. This suggests that application of a cholesterol-lowering strategy prior to cancer development may reduce the risk of advanced PCa. Interestingly, a phase I–II trial testing a high dose (up to 45 mg/kg/day) of lovastatin (a commonly prescribed statin) in patients with CRPC that had failed prior therapies had no effect on the cancer, suggesting that intervention using a cholesterol-based approach was attempted too late for a positive response [69]. Together with our results this prior study suggests that the timing of treatment may be critical, and that application of a cholesterol-lowering strategy once PCa has become aggressive may be too late to prevent or treat CRPC. Although, we have no direct evidence in human patients, our results suggest that cholesterol-lowering therapies might have a particular growth inhibitory effect on early stage prostate tumors that have a positive steroidogenic profile. It remains to be determined if these hypothesized benefits will be achievable in a human patient cohort.

It is important to point out that although we are confident in our analyses, we are reluctant to extrapolate these studies to human patients, as there are substantial differences between our pre-clinical xenograft model and PCas found in human patients: 1) the vascular bed in which the tumor is growing, and thus aspects of angiogenic potential, are different in the subcutaneous flank vs. the prostate; 2) inflammatory processes that likely contribute to PCa growth and aggressiveness are not recapitulated in the xenograft model; 3) tumors do no go through various stages of development from PIN (prostate intraepithelial neoplasia) to frank adenocarcinoma in the xenograft model; and 4) although this limitation is not generally appreciated, the conservation of growth factors, receptors, adhesion molecules etc. is not 100% between mouse and human, and thus certain regulatory factors may not work efficiently in a xenograft model.

In summary, here we demonstrate the novel observation that circulating cholesterol levels influence tumor androgen levels and that pharmacological lowering of cholesterol reduces the level of tumor androgens. Several studies have demonstrated that CRPC tumors can express the full complement of steroidogenic enzymes [49], [66], consistent with the hypothesis that a subset of castration-resistant prostate cancers can carry on *de novo* steroidogenesis. Our findings suggest cholesterol-targeting therapy may inhibit the growth of aggressive prostate cancers via attenuation of intracrine steroidogenesis and, further, that this approach may synergize with therapies directly targeting androgen synthesis and the AR.

## Supporting Information

Figure S1 **Tumor DHT levels in LNCaP xenografts in relation to serum cholesterol levels and tumor weight.** Mean DHT levels (with standard deviation and range) measured by mass spectrometry in tumors from mice grouped by quartile of serum cholesterol (**A**), or in tumors grouped by quartile of tumor weight (**B**). P values from one way ANOVA of mean values in the four quartiles, with a post test for linear trend. The mean cholesterol levels and tumor weights in tumors in each quartile of cholesterol or weight, and the number of mice in each group, are indicated below the x-axis in each graph.
(TIF)
