# Supplementary figures and images for: Impact of Circulating Cholesterol Levels on Growth and Intratumoral Androgen Concentration of Prostate Tumors
Source: PLoS One. 2012 Jan 18;7(1):e30062. doi: 10.1371/journal.pone.0030062 (PMC3261168; doi:10.1371/journal.pone.0030062)

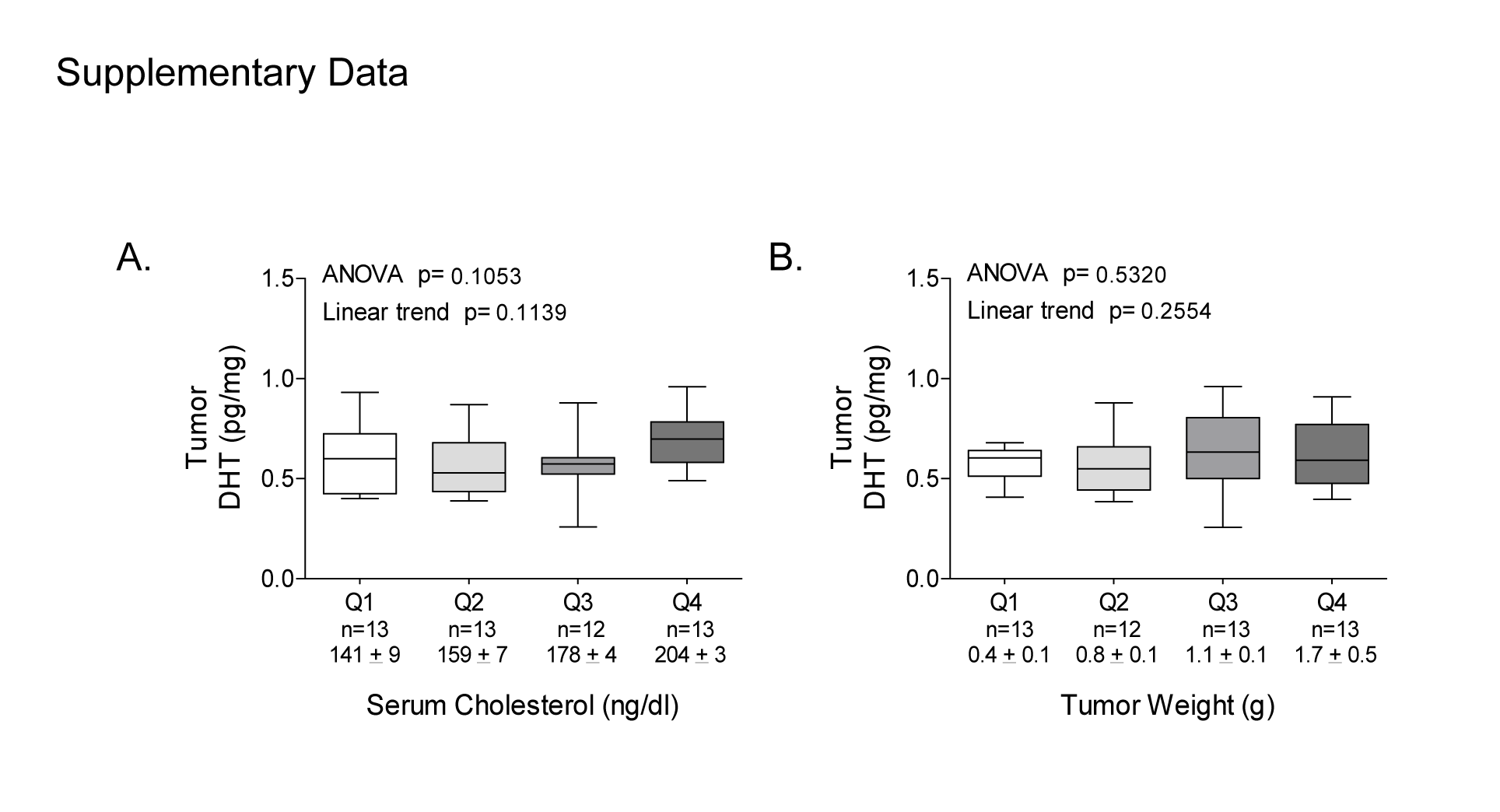

Supplement: Figure S1 — Tumor DHT levels in LNCaP xenografts in relation to serum cholesterol levels and tumor weight. Mean DHT levels (with standard deviation and range) measured by mass spectrometry in tumors from mice grouped by quartile of serum cholesterol (A), or in tumors grouped by quartile of tumor weight (B). P values from one way ANOVA of mean values in the four quartiles, with a post test for linear trend. The mean cholesterol levels and tumor weights in tumors in each quartile of cholesterol or weight, and the number of mice in each group, are indicated below the x-axis in each graph. (TIF) [file pone.0030062.s001.tif]
